# A new species of *Carvalhomiris* from Colombia with an assessment of its phylogenetic position (Heteroptera, Miridae, Orthotylinae)

**DOI:** 10.3897/zookeys.796.22058

**Published:** 2018-11-15

**Authors:** Dimitri Forero, Juanita Rodríguez, Valentina Ocampo

**Affiliations:** 1 Laboratorio de Entomología, Unidad de Ecología y Sistemática, Departamento de Biología, Pontificia Universidad Javeriana, Bogotá, Colombia; 2 Semillero de Entomología, Departamento de Biología, Pontificia Universidad Javeriana, Bogotá, Colombia; 3 Subdirección Científica, Jardín Botánico de Bogotá, Bogotá, Colombia

**Keywords:** Andes, genitalia, Hemiptera, Neotropical region, *Zanchius* group

## Abstract

Plant bugs, species of Miridae (Heteroptera), are not well known in the Neotropics, and Colombia is not an exception. Based on data from the available systematic catalog (Schuh 2002–2013) fewer than 150 species are recorded from the country, clearly an underestimation. Recent fieldwork has resulted in several new interesting taxa from Colombia. *Carvalhomiris* Maldonado & Ferreira, 1971, contains three described species from Colombia and Ecuador. From specimens collected in Jardín, Antioquia, *Carvalhomirishenryi***sp. n.** is described. Images of the dorsal habitus and the male and female genitalia are provided. Based on morphological examination of the new species and published information, morphological characters were coded to construct a phylogenetic matrix for a cladistic analysis in which the phylogenetic position of the new species is assessed. *Carvalhomirishenryi***sp. n.** is the northernmost species of the genus and noteworthy because it is the first record of any species of the genus in the Western Cordillera: all other species are known from the eastern flank of the Andes (Ecuador) or the Eastern Cordillera (Colombia). Natural history observations of the new species, including associations with composites (Asteraceae) are provided. It is speculated that the mirid might be predacious.

## Introduction

Miridae are the most diversified family of Heteroptera with more than 11,000 described species (Ferreira et al. 2015, Wheeler and Krimmel 2015, Henry 2017). In the Neotropics, taxonomic efforts to reveal the family’s diversity were carried out most notably by José Candido de Melo Carvalho and colleagues (Carvalho and Froeschner 1987, 1990, 1994, Henry and Wheeler 1995). Taxonomic knowledge for the family in the Neotropics is still inadequate (Wheeler 2001), partly because the fauna of certain areas is poorly documented. Colombia, for instance, despite being considered a megadiverse country (Mittermeier 1988, Mittermeier et al. 2011, Arbeláez-Cortés 2013), has only about 150 mirid species listed (Schuh 2002–2013), which clearly underestimates the family’s actual diversity.

Among mirid subfamilies, the Orthotylinae comprise six tribes (Schuh 2002–2013), one of which is the paraphyletic Orthotylini (Cassis and Schuh 2012). Within this tribe, Schuh (1974) recognized several informal generic groupings, one of which is the *Zanchius* group, which includes *Carvalhomiris* Maldonado & Ferreira, 1971 (Forero 2009). The generic composition of the *Zanchius* group has varied through time (Schuh 1974, Henry 1995, Forero 2009), but a phylogenetic analysis for the group is not yet available.

*Carvalhomiris* is characterized by an overall pale yellow coloration with greenish areas, usually strong brachypterism in both sexes, and eyes removed from the anterior margin of the pronotum (Maldonado and Ferreira 1971, Forero 2009). The genus has three described species: *C.brachypterus* Maldonado & Ferreira, 1971 and *C.truncatus* Forero, 2009, from three localities in Colombia on the Eastern Cordillera, and *C.bifurcatus* Forero, 2009, from a locality in Ecuador on the eastern flank of the Andes.

Recent fieldwork in Jardín, Antioquia, Colombia, led to the discovery of an additional undescribed species of *Carvalhomiris*. This new species represents the northernmost distribution of the genus and highlights the wide distributional gap among the known species. Additionally, the discovery of this new species allows the putative synapomorphies for *Carvalhomiris* proposed by Forero (2009) to be tested. Therefore, we describe this new species of *Carvalhomiris*, propose the first phylogenetic hypothesis for the genus, and evaluate the phylogenetic position of the new species with respect to previously described species.

## Materials and methods

### Study area and specimens

Specimens were collected near Jardín, Antioquia, Colombia, by beating vegetation along the road. Specimens were mounted, labelled, and deposited in the Entomological collection of the Museo Javeriano de Historia Natural Lorenzo Uribe S.J., of the Pontificia Universidad Javeriana, Bogotá, Colombia (**MPUJ_ENT**) and in the Entomology Collection of the National Museum of Natural History, Smithsonian Institution, USA (**USNM**). Quotation marks were used for specimen data cited verbatim. Different labels were separated by a backslash, with comments placed between square brackets.

### Dissection and terminology

Genitalia were dissected and examined using a Zeiss Discovery V20 stereoscope or a Nikon SMZ1270 stereoscope following Forero (2008). Digital photographs were taken with a Nikon D5300 attached either to a Nikon SMZ1270 stereoscope or a microscope Nikon Eclipse E100. When taking digital photographs using the Nikon SMZ1270 stereoscope, a modified light dome illumination system (Kawada and Buffington 2016) was used. We used a ring LED illumination system from an Olympus stereoscope.

Terminology for male genitalia follows Kelton (1959), Konstantinov (2003), and Forero (2008), except for “vesica”; instead, we refer to the membrane and sclerotizations attached to the phallotheca as endosoma (Cassis 2008, Schwartz 2011); terminology for female genitalia follows Davis (1955), Scudder (1959), and Forero (2008). For the description we assumed that characters not mentioned in the text agree with the generic description of *Carvalhomiris* (Forero 2009).

In proposing a rationale and terminology for the endosomal sclerotizations of male Austromirini, Cassis (2008) designated them as dorsal and ventral endosomal spicules (DES and VES, respectively). Schwartz (2011) expanded the discussion and application of these terms to the Orthotylini, discussing the various configurations of the DES. Schwartz (2011) also noted that several genera of the Orthotylini have a single spiculum that he homologized with the DES. Until further evidence among additional members of the *Zanchius* group is available, we treated the single spiculum of *Carvalhomiris* as the DES, despite not completely agreeing with Cassis (2008) that the base of the DES has an expanded keel.

### Phylogenetic analyses

Based on the taxonomic revision of *Carvalhomiris* (Forero 2009) and the documentation of the new species described herein, morphological characters were proposed and coded to build a matrix with 45 characters (Appendix 1, Suppl. material 1). All characters were treated as non-additive. The characters coded document variation among the species relative to body size, antennal structure and coloration, pronotum structure, hemelytron development and structure, and male and female genitalia. The matrix includes seven terminals. The ingroup comprises the three previously described species of *Carvalhomiris* (Forero 2009) and our species. As outgroups, we included unidentified species of *Parachius* Distant, 1884 and *Itacoris* Carvalho, 1947, both members of the *Zanchius* group (Schuh 1974, Henry 1995, Forero 2009). We also included an additional member of the Orthotylini not closely related to other species of the *Zanchius* group, an unidentified species of *Orthotylus*, which also was used to root the trees.

For the phylogenetic analyses, we used parsimony as the optimality criterion. With the number of terminals very small, an exact solution for the matrix can be provided using a branch-and-bound algorithm (Hendy and Penny 1982, Goloboff et al. 2008). Analyses were carried out using TNT version 1.5 Beta (Goloboff and Catalano 2016).

## Results

### Carvalhomiris henryi sp. n.

Taxon classificationAnimaliaHeteropteraMiridae

http://zoobank.org/EFF8C336-65BF-44E0-A092-CB005AB88FD0

**Figure 1.**
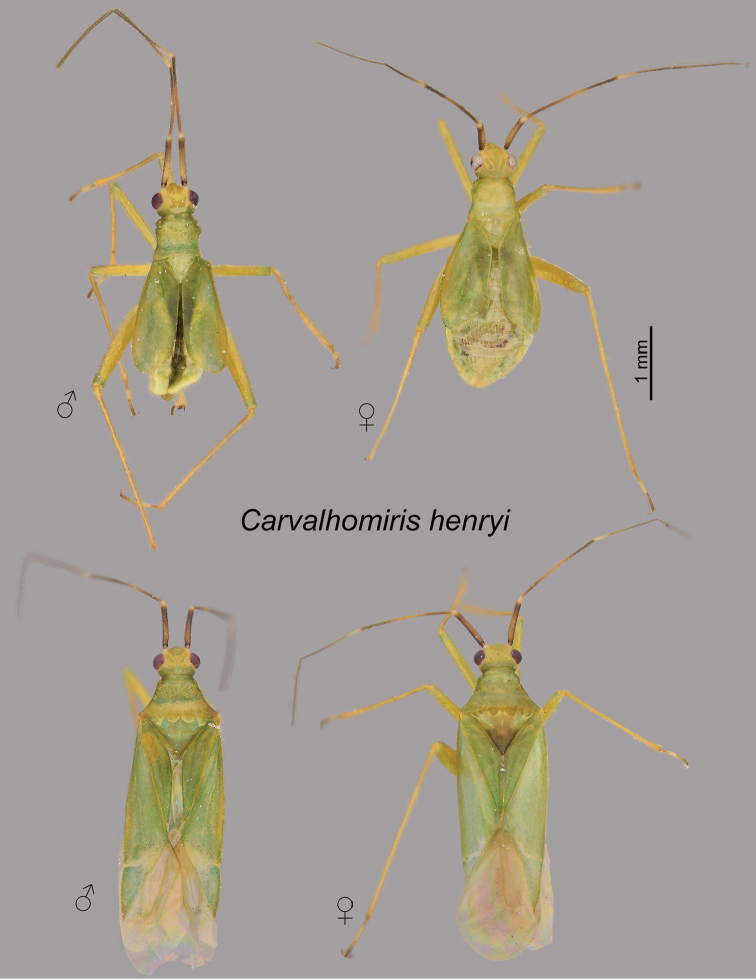
*Carvalhomirishenryi* sp. n. Dorsal habitus images. Male and female brachypterous specimens (upper row), and male and female macropterous specimens (bottom row). The male brachypterous specimen is the holotype.

**Figure 2.**
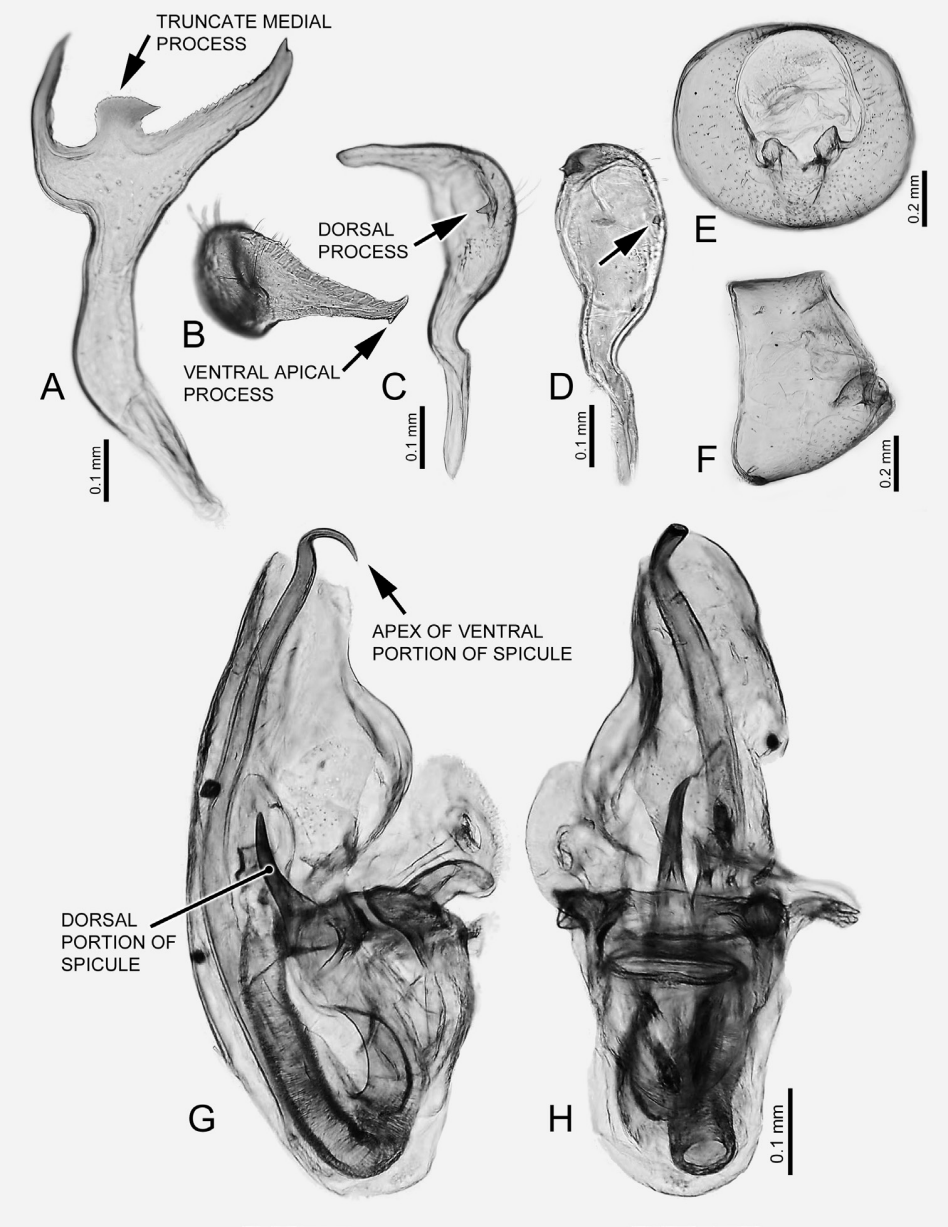
*Carvalhomirishenryi* sp. n. Male genitalia. **A** Right paramere, lateral right view **B–D** left paramere **B** caudal view **C** dorsal view **D** medial view. Arrows on **C** and **D** show the dorsal process **E, F** genital capsule **E** caudal view **F** lateral left view **G, H** aedeagus, not everted **G** lateral right view **H** dorsal view.

**Figure 3.**
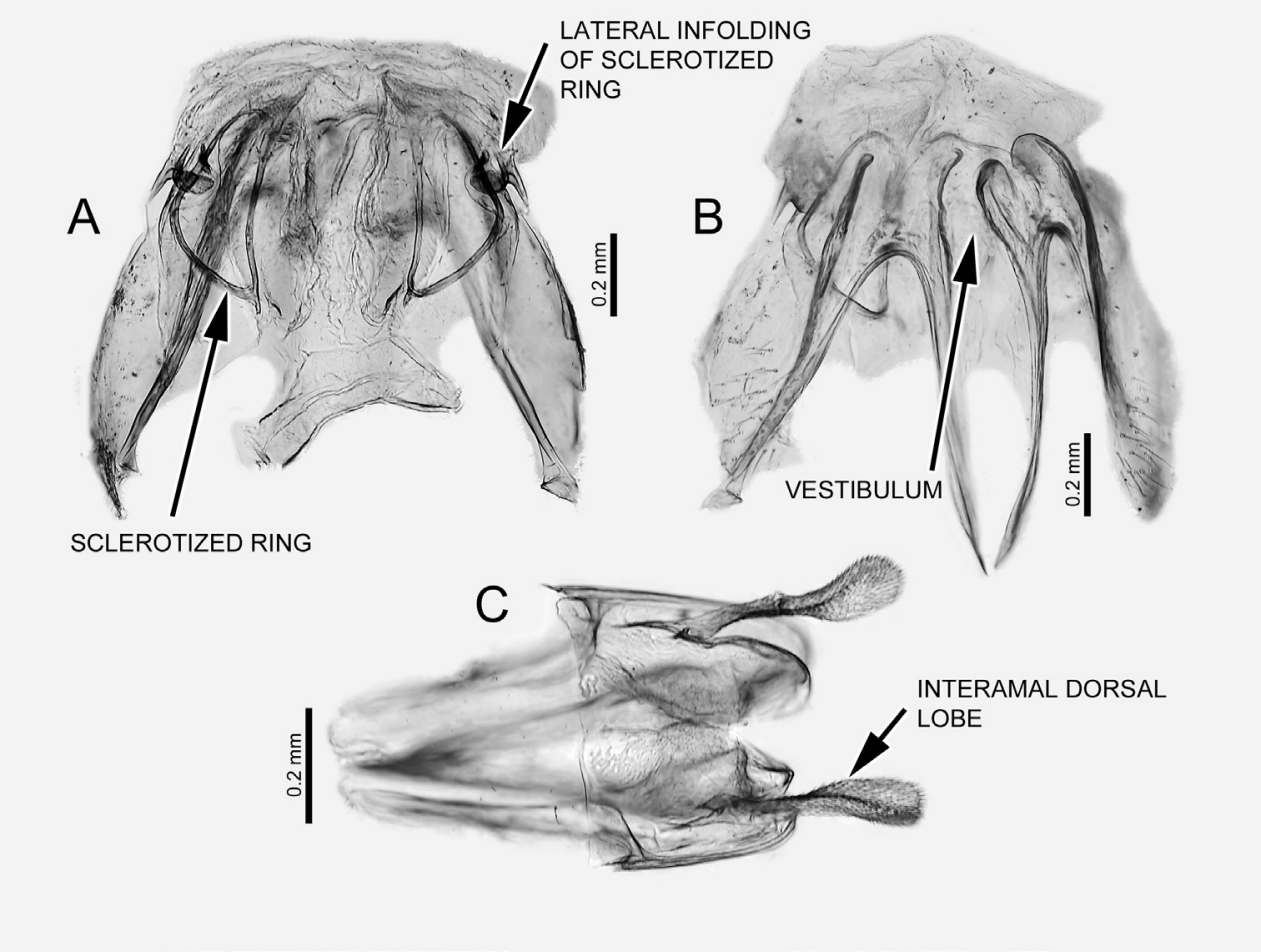
*Carvalhomirishenryi* sp. n. Female genitalia. **A** Dorsal labiate plate and sclerotized rings, dorsal view **B** anterior wall, anterior view, showing the vestibulum, indicating the asymmetrical medial margin of gonapophysis 8 **C** posterior wall, dorsal view, showing the interramal dorsal processes.

**Figure 4.**
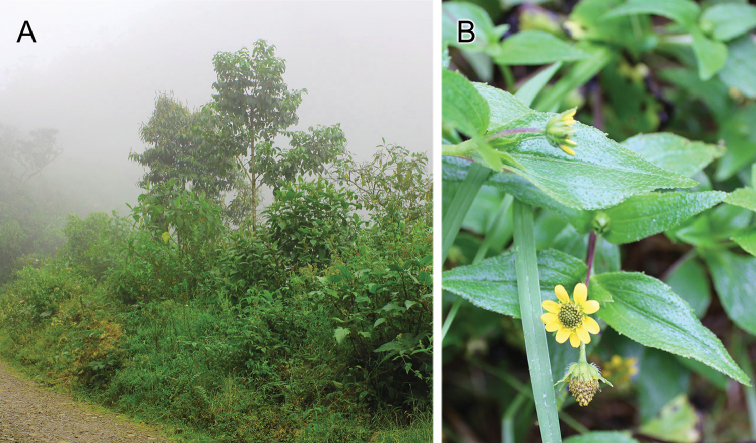
**A** Roadside vegetation in a montane cloud forest on the road to Ventanas, where *C.henryi* sp. n. was found **B***Acmella* sp. (Asteraceae), plant on which specimens of *C.henryi* sp. n. were found.

**Figure 5.**
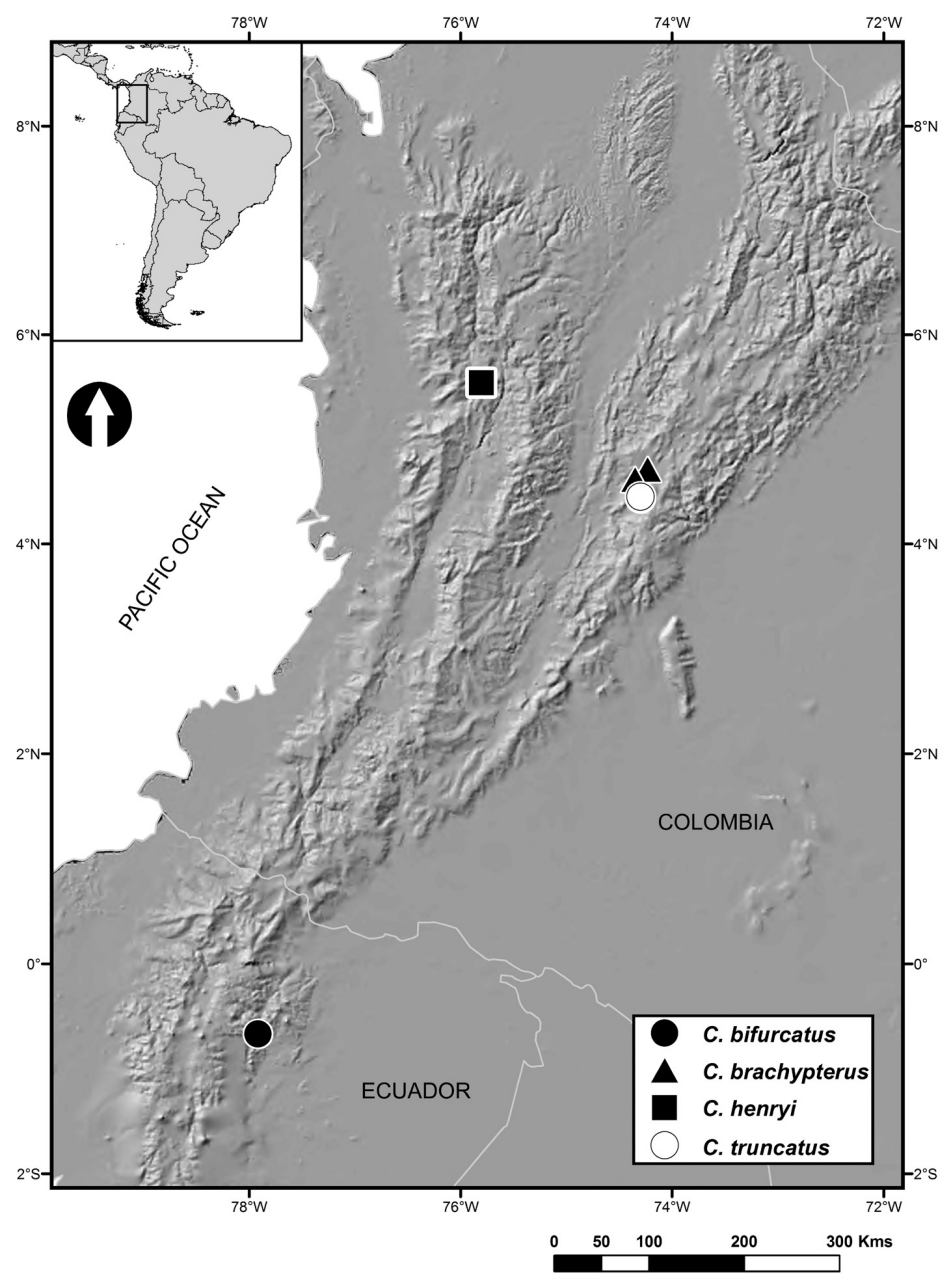
Distribution map of species of *Carvalhomiris*, including *C.henryi* sp. n. (modified from Forero 2009).

Figures 1 , 2 , 3 , 4 , 5

#### Type locality.

COLOMBIA, Antioquia, Jardín, Alto de Ventanas, carretera a Riosucio, 05.5400833°N 75.8035167°W, 2913m, 2 January 2014, D. Forero.

#### Type specimen.

Holotype male, pinned dry brachypterous specimen (figure 1). Original printed labels: “COLOMBIA, Antioquia, Jardín, Alto de Ventanas, carretera a Riosucio, 05.5400833°N 75.8035167°W, 2913m, 2 Ene 2014, D.Forero” / “ex. *Acmella* sp. (Compositae)” / “MPUJ_ENT 0017990” / “♂ Holotype *Carvalhomirishenryi* sp. n. Det. D.Forero 2017” [red printed label] (MPUJ).

#### Paratypes.

2♂ macropters, same data as holotype / MPUJ_ENT 0017988-MPUJ_ENT 0017989 (MPUJ); 10♂ brachypters, same data as holotype / MPUJ_ENT 0017991, MPUJ_ENT 0017993-MPUJ_ENT 0018000 (MPUJ), MPUJ_ENT 0017992 (USNM); 3♀ macropters, same data as holotype / MPUJ_ENT 0018001-MPUJ_ENT 0018002 (MPUJ), MPUJ_ENT 0018003 (USNM); 3♀ brachypters, same data as holotype / MPUJ_ENT 0018005-MPUJ_ENT 0018007 (MPUJ). 3♂ brachypters, Jardín, Alto de Ventanas, carretera a Riosucio, 05.5648333°N 75.7944500°W, 2640m, 2 Ene 2014, D. Forero / MPUJ_ENT 0018013-MPUJ_ENT 0018015 (MPUJ); 5♀ brachypters, same data / MPUJ_ENT 0018008-MPUJ_ENT 0018012 (MPUJ).

#### Other material.

1 nymph, same data as holotype / MPUJ_ENT 0018004 (MPUJ).

#### Diagnosis.

Recognized by the large, basally constricted process on posterior margin of right paramere in males.

#### Description.

*Brachypterous* male. Coloration (Figure 1): Overall coloration pale green. Antennal segments I-IV dark, I dorsomedially pale, II with medial broad pale ring, III basally pale yellow. Head yellowish green, postocular region greenish. Legs yellowish green, third tarsomere dark. Structure (see also Table 1): **Head**: Antennal segment II on medial surface of basal third with decumbent, simple setae. **Thorax**: Pronotum: anterior lobe flat. Hemelytron: Relatively short, not reaching apex of abdomen, apex rounded (Figure 1). **Genitalia**: Genital capsule, large, aperture semicircular (Figure 2E, F). Right paramere with ventral portion straight, directed caudad, apically without spines, posterior margin of paramere medially with truncate, basally constricted process with dorsal portion acutely prolongated (Figure 2A, arrow); left paramere with area caudad to dorsal sensory area with medially projected, acute process of narrow base (Figure 2C, D arrows), apex of paramere on ventral surface with small apical process (Figure 2B, arrow). Aedeagus with right margin of phallotheca with preapical area rounded, smooth (Figure 2G); dorsal endosomal spicule with dorsal portion slightly less than one half length of ventral portion, apex slightly upcurved (Figure 2G), straight in dorsal view (Figure 2H), base relatively narrow (Figure 2G); ventral portion of dorsal endosomal spicule surpassing apex of phallotheca, width homogeneous for most of length, apex strongly hooked with hook curved upwards in lateral view (Figure 2G, arrow) and left in dorsal view (Figure 2H).

*Macropterous male* (Figure 1). Similar to brachypterous male in coloration and structure, but with wider pronotum (Table 1), mesoscutum greatly exposed, and forewings fully developed.

*Brachypterous* female (Figure 1). Similar to brachypterous male in coloration and structure, but slightly larger. **Thorax**: Hemelytron shorter than abdomen, reaching only abdominal segment 5. Genitalia (Figure 3): Dorsal labiate plate on lateral infoldings with small sclerotized area cephalad of sclerotized infolding (Figure 3A). Anterior wall with medial margin of gonapophysis 8 asymmetrical (Figure 3B, arrow), left margin more protuberant than right one, with sclerotized rounded area cephalad of protuberance. Posterior wall as in generic description (Forero 2009), with large, apically expanded interramal dorsal lobes (Fig. 3C).

*Macropterous female* (Figure 1). Similar to brachypterous female in coloration and structure, except forewings fully developed.

**Table 1.** Measurements of *C.henryi* sp. n. Abbreviations are as follows. Ant1: antennal segment 1. Brach: brachypter. Cun: cuneus. Cun-clyp: cuneus-clypeus. IntOcDi: interocular distance. Macr: macropter. Mesoscut: mesoscutellum. Pron: pronotum. Scut: scutellum. NA: not available.

Measurements		Length							Width			
		Total	Cun-Clyp	Head	Pron	Mesoscut	Scut	Cun	Head	Pron	IntOcDi	Ant1
Male brach	Mean	2.96	2.50	0.44	0.55	NA	0.38	NA	0.66	0.72	0.38	0.11
	SD	0.07	0.07	0.05	0.02	NA	0.03	NA	0.03	0.04	0.02	0.02
	Range	0.18	0.18	0.15	0.06	NA	0.06	NA	0.07	0.11	0.04	0.04
	Min.	2.85	2.39	0.36	0.53	NA	0.34	NA	0.61	0.65	0.35	0.09
	Max	3.03	2.57	0.51	0.59	NA	0.40	NA	0.68	0.76	0.39	0.13
Female brach	Mean	2.47	1.85	0.35	0.38	NA	0.35	NA	0.54	0.62	0.30	0.07
	SD	0.04	0.10	0.03	0.01	NA	0.16	NA	0.01	0.06	0.00	0.01
	Range	0.05	0.14	0.04	0.02	NA	0.23	NA	0.01	0.08	0.00	0.01
	Min.	2.44	1.78	0.33	0.37	NA	0.23	NA	0.53	0.58	0.30	0.06
	Max	2.49	1.92	0.37	0.39	NA	0.46	NA	0.54	0.66	0.30	0.07
Male macr	Mean	4.51	3.73	0.30	0.48	0.30	0.37	0.78	0.65	1.06	0.37	0.09
	SD	0.01	0.05	0.08	0.10	0.09	0.02	0.02	0.01	0.01	0.04	0.01
	Range	0.02	0.07	0.12	0.14	0.13	0.03	0.03	0.01	0.02	0.05	0.01
	Min.	4.50	3.69	0.24	0.41	0.23	0.35	0.76	0.64	1.05	0.34	0.08
	Max	4.52	3.76	0.36	0.55	0.36	0.38	0.79	0.65	1.07	0.39	0.09
Female macr	Mean	4.50	3.73	0.41	0.53	0.26	0.36	0.74	0.63	1.06	0.38	0.09
	SD	0.26	0.29	0.07	0.01	0.00	0.03	0.06	0.00	0.07	0.02	0.04
	Range	0.37	0.41	0.10	0.02	0.00	0.04	0.08	0.00	1.04	0.37	0.07
	Min.	4.31	3.52	0.36	0.52	0.26	0.34	0.70	0.63	0.07	0.02	0.04
	Max	4.68	3.93	0.46	0.54	0.26	0.38	0.78	0.63	1.11	0.39	0.11

#### Etymology.

We are pleased to dedicate this new species to our friend and colleague Thomas J. Henry. The new name is treated as a Latin noun in the genitive case. Tom is an indefatigable entomologist, always willing to share his knowledge and help others. Tom’s enthusiasm for Heteroptera is contagious, not only in the lab but also in the field. Tom collected a long series of specimens of *Carvalhomiris* in Ecuador in 1996 that was described as *C.bifurcatus* (Forero 2009); therefore, we take great pleasure in dedicating this new Colombian species of *Carvalhomiris* to Tom.

#### Distribution.

Known from the type locality on a road near Jardín, Antioquia, in the western Cordillera in Colombia. It was found from 2640 to 2913 meters in elevation, in a high Andean cloud forest (Figure 4A). This locality is the northernmost for any species of the genus (Figure 5).

#### Plant associations.

Specimens of the new species were mostly associated with small roadside herbs of *Acmella* sp. (Asteraceae) (Figure 4B). Despite multiple observations, no direct plant feeding was observed.

## Discussion

Species of *Carvalhomiris* are rather homogeneous in external morphology, but their male genitalia are distinct (Forero 2009). *Carvalhomirishenryi* sp. n. is more closely related to *C.brachypterus* and *C.truncatus*, than to *C.bifurcatus* based on characters of the male and female genitalia (see also phylogenetic analysis below). *Carvalhomirishenryi* sp. n. is similar to *C.truncatus* in having a medial process on the posterior margin of the paramere, but this process in *C.henryi* sp. n. is basally constricted, whereas in *C.truncatus* it is not. *Carvalhomirishenryi* sp. n. is similar to *C.brachypterus* in structure of the left paramere with an acute, medially directed, dorsal process caudad of the sensory area; in *C.henryi* sp. n. this process is large, not small as in *C.brachypterus*, and differs from that in *C.truncatus* which is large and broad. The structure of the dorsal endosomal spicule differentiates *C.henryi* sp. n. from its congeners. These characters help to identify this new species, but its mixed character associations preclude an unambiguous assessment of phylogenetic relationships.

Heteropteran distributions have been little documented in Colombia, especially for geographically restricted taxa such as species of *Carvalhomiris* in the high Andes. An increased knowledge of biogeographic patterns might reveal diversification processes that produced these distributional ranges. Unknown is what is driving speciation processes in the Andes, a question that relates to the natural history of *Carvalhomiris*. It is not certain that species of this genus are strictly phytophagous. They might be omnivores, as is the case in certain orthotylines (Wheeler 2001). To begin answering these questions, further fieldwork is requisite. Because *C.henryi* sp. n. is known from a different cordillera than its Colombian congeners, the discovery of new species between the known localities can be anticipated. In addition, little-known habitats in Colombia above 2400 m should be thoroughly explored to attain a more complete panorama of the evolution of the group.

The collection of *C.henryi* sp. n. from a composite, *Acmella* sp., represents the second plant association for a species of *Carvalhomiris*. Feeding habits of the new species are unknown, but it might not be strictly phytophagous. The family Tropaeolaceae, on which *C.truncatus* has been found (Forero 2009), is not closely related to the Asteraceae. Although *C.henryi* sp. n. was most numerous on *Acmella*, it also was taken on other plants. This orthotyline might remain on *Acmella* before dispersing to other plant species when the quality of its typical host deteriorates or when prey numbers decline. The documented associated plants in *Carvalhomiris* are not closely related. For example, *C.truncatus* is associated with *Tropaeolum* sp. (Tropaeolaceae) (Forero 2009), a family far removed from the Asteraceae. Specimens of *C.henryi* sp. n. were collected on vegetation along the road, and despite being more abundant on *Acmella*, they were found on other plants in lesser numbers. We suggest that species in *Carvalhomiris* might be predators, or at least facultatively predaceous. In searching for prey, phytophagous insects can be found readily and in abundance on their host plants. The prey of predatory species, and their associated plants, can vary, which also might be the case in other members of the *Zanchius* group.

### Key to the species of *Carvalhomiris* (modified from Forero 2009)

**Table:** 

1	Right paramere with ventral portion greatly elongated and apically bifurcated; brachypterous male and female with apex of hemelytron acute; medial margin of first gonapophysis nearly symmetrical	***bifurcatus* Forero, 2009**
– R	ght paramere neither greatly elongated nor strongly bifurcated on ventral portion; brachypterous male and female with apex of hemelytron rounded; anterior medial margin of first gonapophysis strongly asymmetrical	**2**
2	Posterior serrated margin of right paramere nearly straight	***brachypterus* Maldonado & Ferreira, 1972**
– P	sterior serrated margin of right paramere with a median process	**3**
3	Median process of posterior margin of right paramere large, nearly truncate, with wide base; dorsal portion of endosomal spicule with apex strongly left-curved; dorsal process of left paramere short, base wide	***truncatus* Forero, 2009**
– M	dian process of posterior margin of right paramere small, constricted basally (Fig. 2A, arrow); dorsal portion of endosomal spicule with apex straight (Fig. 2H); dorsal process of left paramere long and acute, base narrow (Fig. 2C, 2D, arrows)	***henryi* sp. n.**

### Phylogenetic analysis

**Figure 6.**
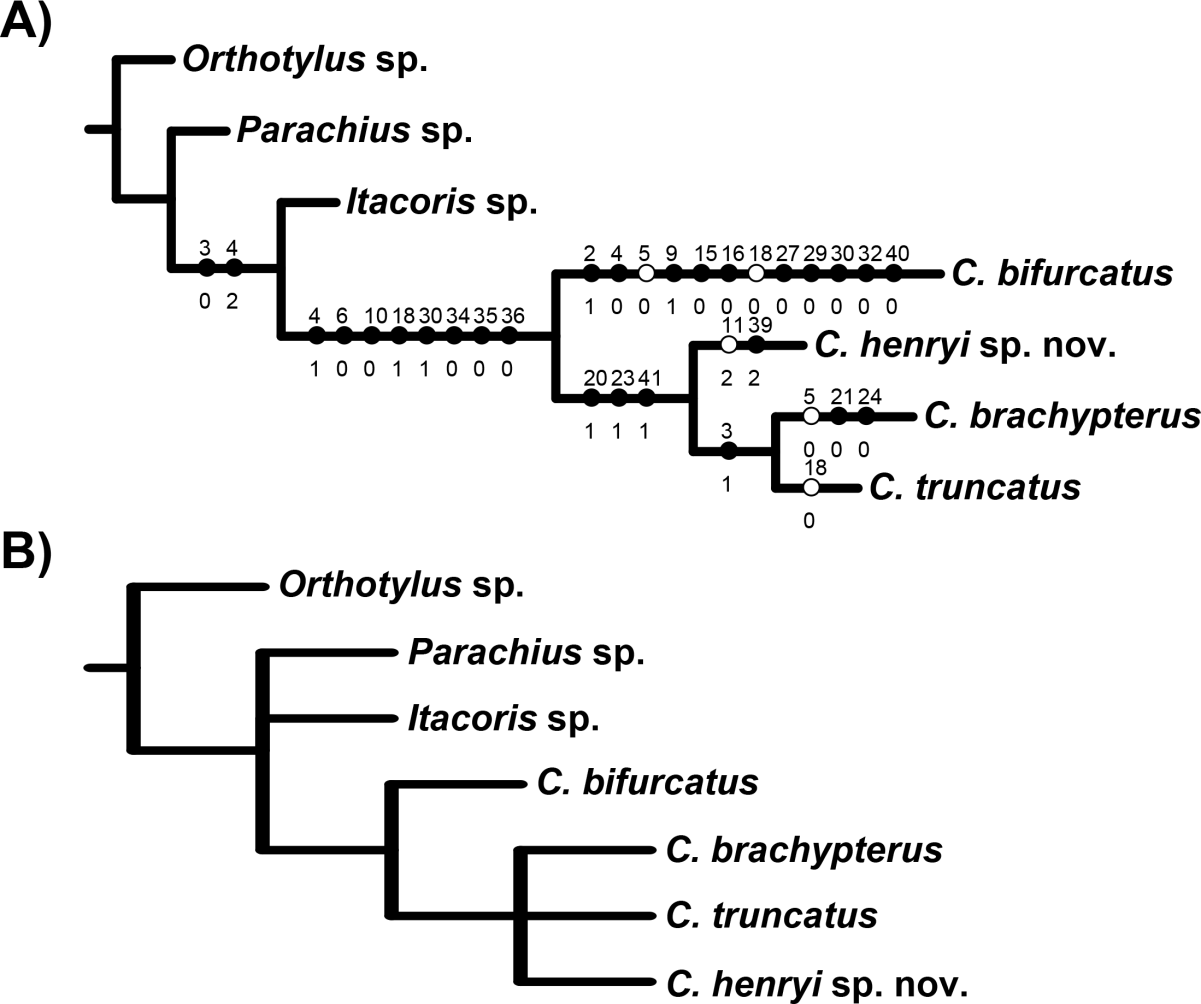
Phylogenetic analysis of *Carvalhomiris* species, including *C.henryi* sp. n. **A** One of the two trees obtained (Ci: 81, Ri: 67) with characters and character states indicated on the branches **B** consensus tree of the two trees obtained.

The implicit enumeration search strategy for the phylogenetic analysis resulted in two equally parsimonious trees (Figure 6A; Ci: 81, Ri: 67). The strict consensus showed that either *Itacoris* or *Parachius* could be considered the sister group of *Carvalhomiris* depending on the topology chosen. The strict consensus also showed that *Carvalhomiris* is monophyletic, but that their internal relationships are not completely resolved. *Carvalhomirishenryi* sp. n. is placed in a polytomy with *C.brachypterus* and *C.truncatus*, with *C.bifurcatus* as the sister group of this clade. These associations not only show the similarities among the three species, but also point to the particular apomorphies exhibited by *C.bifurcatus*. *Carvalhomirisbifurcatus* is also the southernmost species of the genus (Ecuador); it is not known if there is a particular character transformation scheme in relation to geography. The resulting phylogenetic hypotheses do not allow a clear-cut interpretation of a geographic pattern within the *C.brachypterus* clade. Even though geographically close species also might be expected to be phylogenetically close, the recovered pattern was ambiguous in this respect. The further description of new species of *Carvalhomiris* might resolve this ambiguity.

The monophyly of *Carvalhomiris* was suggested without the testing of a cladistic hypothesis, but a few putative synapomorphies were noted (Forero 2009). An evaluation of these putative characters in our phylogenetic analysis enables most of the previously suggested synapomorphies to be corroborated: strong brachyptery in both sexes; large genital capsule in males with the anterolateral margin strongly curved; dorsal endosomal spicule C-shaped inserted near the base of the aedeagus; dorsal labiate plate with a median dorsal depression; and lateral infoldings of the sclerotized rings with a caudad acute process. The following were also proposed as putative synapomorphies (Forero 2009), but were not recovered as such in this analysis: right paramere apically expanded, apical margin of paramere serrated, and endosoma with a single spicule.

Further collecting in the wide geographic gap between the known localities of *Carvalhomiris* species might reveal additional species and allow further testing of phylogenetic relationships in the genus.

## Supplementary Material

XML Treatment for Carvalhomiris henryi

## Appendix 1

Morphological character list and their character states used in the phylogenetic analysis of *Carvalhomiris* species.

0. Relative body size: small (0); medium (1).

1. Antenna, type of setae on segment II medially on basal third: suberect (0); decumbent (1).

2. Antenna, length of setae on segment II medially on basal third: short (0); very short (1).

3. Antennal segment II, coloration: dark brown, pale median band (0); apical half brown, basal half pale (1); dark brown (2).

4. Antennal segment III, coloration: brown, base pale (0); brown, basal half pale yellow (1); brown, basal third pale (2); dark brown (3).

5. Pronotum, anterior lobe, structure of surface: slightly convex (0); flat (1).

6. Hemelytron development: strong brachypterism in both sexes (0); macropterous in both sexes (1).

7. Hemelytron in brachypterous specimen, relative length with respect to abdomen: short, not reaching genital capsule (0); long, reaching genital capsule (1).

8. Hemelytron in brachypterous specimen, shape of apex: acute (0); rounded (1).

9. Hemelytron, density of setae on apex (or cuneus): sparse (0); abundant (1).

10. Genital capsule, relative size to abdomen length: about half abdomen (0); about 2/3 abdomen (1); about 1/3 abdomen (2).

11. Genital capsule, structure of cephalic lateral margin in lateral view: strongly curved (0); straight (1); slightly curved (2).

12. Genital capsule, extent of ventral posterior margin relative to insertion of parameres: not projected (0); greatly projected (1).

13. Right paramere, structure of distal half: clubbed (0); nearly cylindrical (1).

14. Right paramere, structure apex: without a differentiated ventral portion (0); with a differentiated ventral portion (1).

15. Right paramere, structure of ventral portion: strongly curved dorsally (1); straight, directed caudad (1); straight, directed ventrad (2).

16. Right paramere, relative length of ventral portion: as long as apical margin (0); about half as long as apical margin (1).

17. Right paramere, structure of apical ventral portion: with a preapical spine (0); strongly bifurcated (1); as an acute process (2).

18. Right paramere, ornamentation of apical margin: gently serrate (0); strongly serrate (1); smooth, not serrate (2).

19. Right paramere, orientation of apical margin: vertical (0); reclined (1).

20. Right paramere, structure of apical margin: entire, uninterrupted (0); interrupted (1).

21. Right paramere, structure of process on apical margin interrupting margin: medium-sized spine near dorsal portion (0); large, median truncate process (1).

22. Right paramere, base of process on apical margin: constricted (0); not constricted (1).

23. Left paramere, structure of area caudad to dorsal sensory area: flat (0); with a dorsal process (1).

24. Left paramere, relative size of base of dorsal process caudad to dorsal sensory area: narrow (0); broad (1).

25. Left paramere, structure of apex: deeply cleft (0); not cleft (1).

26. Left paramere, structure on ventral surface: flat, not produced (0); with a ventral process (1).

27. Left paramere, size of apical process on ventral surface: large (0); small (1).

28. Left paramere, structure of apical process on ventral surface: acute (0); rounded (1).

29. Left paramere, structure of preapical ventral area: protruding (0); nearly flat (1).

30. Left paramere, cuticle on preapical ventral area: with medium-sized trichia (0); with very small trichia (1); flat, without trichia (2).

31. Phallotheca, structure of basal lateral area with flaplike protuberances: flat (0); with paired protuberances (1).

32. Phallotheca, structure of preapical margin: acute (0); broadly rounded (1).

33. Endosoma, number of spicules: one (0); two (1); three (2).

34. Endosomal spicule DES2, shape of spicule in lateral view: C-shaped (0); straight (1).

35. Endosomal spicule DES2, structure of the base: wide (0); narrow (1).

36. Endosomal spicule DES2, relative position in repose with respect to cephalad portion of aedeagus: close to cephalad portion (0); barely reaching caudal portion of articulatory apparatus (1).

37. Endosomal spicule DES2, C-shaped, dorsal portion, length with respect to ventral portion: three fourths of the length (0); half the length (1).

38. Endosomal spicule DES2, C-shaped, ventral portion, structure of apex: acute (0); expanded (1).

39. Endosomal spicule DES2, C-shaped, ventral portion, structure of apex: straight (0); curved dorsally (1); curved cephalad (2).

40. Endosomal spicule DES2, relative length with respect to apex of phallotheca: not reaching apex (0); reaching or surpassing apex (1).

41. First gonapophysis, symmetry of anterior medial margin: nearly symmetrical (0); strongly asymmetrical (1).

42. Dorsal labiate plate, structure of area of insertion of accessory gland: produced ventrally as a conical depression (0); flat (1).

43. Sclerotized rings of dorsal labiate plate, lateral structure: with lateral infoldings (0); without lateral infoldings (1).

44. Sclerotized rings of dorsal labiate plate, lateral infoldings structure of caudal margin: with acute process (0); rounded (1).

**Table A1.** Matrix of morphological characters and their states used in the phylogenetic analysis of *Carvalhomiris* species ("–": inapplicable; "?": unknown).

**Taxon**	**Characters**																						
	**0**	**1**	**2**	**3**	**4**	**5**	**6**	**7**	**8**	**9**	**10**	**11**	**12**	**13**	**14**	**15**	**16**	**17**	**18**	**19**	**20**	**21**	**22**
*Orthotylus* sp.	1	1	0	2	3	–	1	–	–	0	1	2	1	1	0	–	–	–	–	–	–	–	–
*Itacoris* sp.	0	0	0	0	2	1	1	–	–	0	2	1	1	0	1	1	1	0	2	1	0	–	–
*Parachius* sp.	0	1	0	2	3	1	1	–	–	1	2	1	0	0	1	2	1	2	2	0	0	–	–
* C. bifurcatus *	0	0	1	0	0	0	0	1	0	0	0	0	0	0	1	0	0	1	0	1	0	–	–
* C. brachypterus *	0	1	0	1	1	0	0	0	1	0	0	0	0	0	1	1	1	0	1	0	1	0	–
* C. truncatus *	0	1	0	1	1	1	0	0	1	0	0	0	0	0	1	1	1	0	0	0	1	1	1
*C.henryi* sp. n.	0	1	0	0	1	1	0	0	1	0	0	2	0	0	1	1	1	0	1	0	1	1	0
	**23**	**24**	**25**	**26**	**27**	**28**	**29**	**30**	**31**	**32**	**33**	**34**	**35**	**36**	**37**	**38**	**39**	**40**	**41**	**42**	**43**	**44**	
*Orthotylus* sp.	0	–	0	0	–	–	1	2	2	1	2	1	1	1	–	–	–	1	0	1	0	1	
*Itacoris* sp.	0	–	1	0	–	–	1	2	0	1	1	1	1	1	–	–	–	1	?	?	?	?	
*Parachius* sp.	0	–	1	1	1	1	1	2	0	1	0	1	1	1	–	–	–	1	0	1	0	1	
* C. bifurcatus *	0	–	1	1	0	0	0	0	0	0	0	0	0	0	0	0	0	0	0	0	0	0	
* C. brachypterus *	1	0	1	1	1	1	1	1	0	1	0	0	0	0	1	1	1	1	1	0	0	–	
* C. truncatus *	1	1	1	1	1	1	1	1	0	1	0	0	0	0	1	1	1	1	1	0	0	–	
*C.henryi* sp. n.	1	1	1	1	1	0	1	1	0	1	0	0	0	0	1	1	2	1	1	0	0	–	

## Supplementary materials

Supplementary material 1 Carvalhomiris matrix (phylogenetic) Data type: phylogenetic data This is an open access article distributed under the terms of the Creative Commons Attribution License (CC BY 4.0), which permits unrestricted use, distribution, and reproduction in any medium, provided the original author and source are credited.Dimitri Forero, Juanita Rodríguez, Valentina Ocampo
